# Determinants of digital health literacy among older adult patients with chronic diseases: a qualitative study

**DOI:** 10.3389/fpubh.2025.1568043

**Published:** 2025-03-31

**Authors:** Yujiao Shao, Xiumu Yang, Qin Chen, Hongyan Guo, Xiaocui Duan, Xuejun Xu, Jianxing Yue, Zeyu Zhang, Shuang Zhao, Shiqing Zhang

**Affiliations:** ^1^School of Nursing, Bengbu Medical University, Bengbu, Anhui, China; ^2^General Practice Education Development Research Center, Bengbu Medical University, Bengbu, Anhui, China; ^3^Nursing Department, Suzhou Hospital Affiliated with Anhui Medical University, Suzhou, Anhui, China; ^4^Department of General Practice, First Affiliated Hospital of Bengbu Medical University, Bengbu, Anhui, China; ^5^Department of Rehabilitation Medicine, Second Affiliated Hospital of Bengbu Medical University, Bengbu, Anhui, China; ^6^Nursing Department, No. 902 Hospital of the People’s Liberation Army Joint Logistics and Security Force, Bengbu, Anhui, China; ^7^Nursing Department, First People's Hospital Affiliated with Bengbu Medical University, Bengbu, Anhui, China

**Keywords:** older adult, chronic diseases, digital health literacy, determinants, qualitative research

## Abstract

**Objective:**

This study aimed to identify the factors influencing digital health literacy in older adult patients with chronic diseases.

**Methods:**

A descriptive qualitative approach incorporated purposive and snowball sampling methods. Semi-structured interviews were conducted with 32 older adult patients with chronic diseases from three hospitals in Anhui Province between October 2023 and May 2024. Data were coded and analyzed using Nvivo 12.0 software and content analysis.

**Results:**

Two main themes and nine subthemes emerged: driving factors: these include the accessibility of digital health resources, perceived value and management needs, family economic and social benefits, and social network support systems. Restricting factors: These include cognitive blind spots and understanding biases, basic skills and challenges in digital adaptation, psychosocial limitations, issues with health information quality, and concerns about digital security risks.

**Conclusion:**

The digital health literacy of older adult patients with chronic diseases is generally low, characterized by cognitive blind spots, and influenced by various personal and social factors. It is recommended to engage social forces, optimize the accessibility and applicability of digital health resources, create a supportive digital health environment, and help older adult patients improve their digital health literacy to enhance chronic disease self-management through digital health technology.

## 1 Introduction

By the end of 2023, China’s population aged 60 and above reached 297 million, accounting for 21.1% of the total population. Among them, 217 million were aged 65 and above, comprising 15.4% of the total population (1). This proportion is expected to rise to 30% by 2050 (2), highlighting the deepening trend of population aging in China (3). As aging intensifies, the prevalence of chronic non-communicable diseases (NCDs) continues to grow. NCDs, characterized by high prevalence, long-term disease progression, suboptimal disease management outcomes, and substantial economic burden, pose significant threats to public health and socio-economic development. Globally, NCDs cause approximately 41 million deaths annually, accounting for 74% of all deaths (4). In China, over 80% of deaths are attributed to NCDs. Older adult individuals with NCDs often face high prevalence rates, multiple comorbidities, mental health challenges, and increased disability and mortality rates, complicating self-management.

Chronic disease self-management involves patients adopting self-management strategies under healthcare professional guidance to control disease progression. This includes preventive and therapeutic behaviors to address the physical and emotional challenges of NCDs in daily life (5). Effective chronic disease self-management can prevent disease deterioration and complications. The demand for telemedicine, health management, and personalized medical services among older adult patients with NCDs is steadily increasing. However, China continues to face significant challenges in preventing, treating, and managing NCDs (3, 6).

Amid global digitalization, the World Health Organization (WHO) published the Global Strategy on Digital Health (2020–2025), establishing digital health governance as a global priority (7). China’s healthcare services are transitioning toward digital health (8, 9). Digital health technologies enhance health management, advance personalized medicine, and facilitate health information exchange. However, as digital health technologies evolve, they create opportunities to address aging-related challenges while increasing demands on older adult patients’ self-management. Digital health literacy is the ability to obtain, assess, use, and communicate health information through digital technologies. It plays a crucial role in health promotion and disease management (10, 11).

Despite advancements in digital healthcare, access remains unequal. Digitally disadvantaged populations struggle to access these services, worsening health disparities (12, 13). International research has identified factors such as gender, age, education, technology experience, location, and health status as key influences on digital health literacy (14). Higher digital health literacy correlates with better quality of life in heart failure patients (15). Qualitative studies reveal older adult individuals’ concerns about digital health, including technological adaptability, information credibility, cost, motivation, and telemedicine feasibility (16). Recent digital health literacy research has shifted from individual skills to social and organizational influences, highlighting its role in health equity (17).

In China, digital health literacy research emerged later, initially focusing on quantitative studies and tool development (10, 18, 19). Studies have focused on students (20), the general public (21), pregnant women (22), the older adults (23–25), and chronic disease patients (26). Studies show that older adult individuals in China have low digital health literacy limited digital health adoption, and poor usage outcomes, creating barriers to healthcare integration (10, 23, 24). Limited research on digitally disadvantaged older adult chronic disease patients hinders understanding of digital health literacy influences and mechanisms. Digital health literacy determinants vary across socio-cultural contexts.

Qualitative research systematically explores individuals’ lived experiences through textual narratives. It uses an inductive approach, focusing on holism, deep understanding, individuality, and social-psychological meaning (27). The Social-Ecological Systems Theory (SET) (28, 29) posits that individual capabilities develop through interactions within environmental systems: the microsystem (personal factors), mesosystem (social support), and macrosystem (community and policy).

This study applies qualitative research within SET to examine digital health literacy determinants and mechanisms among older adult chronic disease patients in China at individual, interpersonal, and societal levels. Findings will inform evidence-based intervention strategies.

## 2 Participants and methods

### 2.1 Participants

This study used purposive sampling combined with snowball sampling to recruit older adult patients with chronic diseases from three hospitals in Anhui Province between October 2023 and May 2024. The inclusion criteria were: (1) age ≥ 60 years; (2) a confirmed chronic disease diagnosis based on ICD-10 criteria; including but not limited to hypertension, diabetes mellitus, coronary artery disease, cancer, chronic obstructive pulmonary disease, and osteoarthritis; (3) own or have access to a digital device (e.g., accessing the internet via a smartphone) and be able to use it independently or with assistance from others; (4) clear consciousness and normal communication or reading abilities; (5) informed consent and willingness to participate. Exclusion criteria included: (1) severe cognitive, linguistic, auditory, or mental impairments; (2) being in an acute phase of a major illness or critical condition with severe complications; (3) extended or difficult communication issues; (4) concurrent participation in other studies. This study followed the principle of maximum variation sampling, selecting participants based on diverse factors, including age, gender, residence, education level, number of chronic diseases, perception of digital health information, and scores on the Digital Health Literacy Assessment Scale (10). This approach ensured sample representativeness and enhanced diversity.

The sample size was determined using the principles of data adequacy and information saturation, assessed through both data and theoretical saturation. Data saturation was reached when no new information emerged, interview content became repetitive, and similar responses were frequently observed, indicating that further data collection was unnecessary (30). Theoretical saturation occurred when no new themes or insights emerged, and all relevant concepts, attributes, and interrelationships had been thoroughly explored, suggesting additional data would not contribute to new theoretical understanding (31). Throughout the study, the research team continuously analyzed interview data to assess saturation. After the 30th interview, no new information or concepts emerged, confirming data saturation. To further validate this, two additional interviews were conducted. In total, 32 patients participated in the study. Data were collected through face-to-face semi-structured interviews, and participants were assigned unique identifiers (N1–N32) in accordance with privacy protection guidelines.

Detailed demographic information is provided in Table 1. Among the 32 patients, hypertension was the most common chronic disease, accounting for the highest proportion. The differences in chronic disease types were statistically significant (χ^2^ = 73.71, df = 10, *p* < 0.0001; Table 2). Smartphones were the most widely used digital devices, representing the dominant choice. The differences in the number of digital devices used were also statistically significant (χ^2^ = 66.71, df = 7, *p* < 0.0001; Table 3). The study was approved by the Ethics Committee of Bengbu Medical University (Approval No. [2023]369). All participants voluntarily participated and signed informed consent forms.

**Table 1:** General information of older adult patients with chronic diseases (*n* = 32).

Number	Age	Gender	Educational level	Living situation	Place of Residence	Number of chronic diseases	Duration of illness^a^ (Years)	Number of Medications	Average daily internet usage (Hours)	Perceived digital health information				Digital health literacy Score^b^
										Usefulness	Ease of Use	Risk	Trustworthiness	
1	60	Female	Middle school	Spouse	City	1	3–5	1–3	>5	Very useful	Very easy	Very Low	Very Untrustworthy	70
2	69	Female	Vocational school	Spouse	City	3	>10	1–3	<1	Average	Quite easy	Average	Average	61
3	74	Male	High school	Spouse	City	2	>10	4–6	2–3	Quite useful	Very easy	Very Low	Very Untrustworthy	61
4	62	Male	Middle school	Spouse	City	1	>10	1–3	1–2	Very useful	Very easy	Very Low	Very Untrustworthy	56
5	62	Male	University	Spouse	City	3	>10	4–6	1–2	Quite useful	Quite easy	Quite Low	Average	48
6	74	Female	University	Alone	City	1	>10	1–3	2–3	Average	Average	Quite High	Not Very Trustworthy	37
7	68	Female	High school	Spouse	City	2	>10	1–3	2–3	Quite useful	Quite easy	Average	Average	57
8	72	Female	Primary school	children	City	2	>10	1–3	<1	Quite useful	Quite easy	Average	Quite Trustworthy	49
9	78	Male	High school	Spouse	City	1	>10	1–3	2–3	Average	Quite easy	Quite Low	Average	45
10	77	Female	college	Spouse	City	2	1–3	None	1–2	Average	Quite easy	Very Low	Not Very Trustworthy	52
11	60	Female	Middle school	Spouse	City	1	1–3	None	3–4	Quite useful	Quite easy	Quite Low	Quite Trustworthy	46
12	62	Male	Middle school	Spouse	City	2	1–3	1–3	2–3	Average	Quite easy	Quite Low	Not Very Trustworthy	47
13	79	Male	University	Spouse	City	2	>10	1–3	2–3	Quite useful	Quite easy	Average	Quite Trustworthy	56
14	60	Male	college	Alone	City	2	1–3	1–3	2–3	Average	Very easy	Average	Average	55
15	63	Male	High school	Spouses and children	Town	1	<1	None	3–4	Average	Quite difficult	Quite High	Quite Trustworthy	62
16	73	Female	Middle school	Spouse	City	1	1–3	1–3	1–2	Very useful	Average	Average	Very Untrustworthy	52
17	60	Male	Primary school or below	Spouses and children	City	1	<1	None	3–4	Quite useful	Quite easy	Quite Low	Quite Trustworthy	48
18	60	Female	Primary school	Alone	City	1	1–3	None	2–3	Average	Quite easy	Quite High	Average	59
19	76	Male	Middle school	Spouse	City	2	>10	1–3	>5	Average	Average	Quite High	Average	53
20	64	Female	Primary school	Alone	City	2	<1	1–3	>5	Quite useful	Average	Quite High	Quite Trustworthy	63
21	60	Male	Graduate degree	Spouse	Town	1	5–10	None	4–5	Very useful	Very easy	Very Low	Very Untrustworthy	48
22	61	Female	Below primary school	Mother and child	City	2	>10	1–3	>5	Quite useful	Average	Average	Quite Trustworthy	27
23	82	Male	University	Spouse	City	3	1–3	1–3	4–5	Average	Average	Average	Average	69
24	69	Male	High school	Spouse	Rural	3	1–3	1–3	2–3	Quite useful	Quite easy	Quite Low	Quite Trustworthy	49
25	60	Male	University	Spouse	City	3	3–5	1–3	3–4	Quite useful	Very easy	Very Low	Very Untrustworthy	53
26	60	Male	Middle school	Children	City	2	>10	1–3	1–2	Quite useful	Quite easy	Quite Low	Quite Trustworthy	65
27	60	Female	Middle school	Spouses and children	City	2	3–5	None	3–4	Very useful	Very easy	Quite Low	Quite Trustworthy	54
28	64	Male	Middle school	Alone	City	1	>10	1–3	3–4	Average	Quite difficult	Quite High	Average	54
29	62	Male	University	Spouse	City	3	>10	None	3–4	Not useful	Very easy	Very High	Very Untrustworthy	48
30	62	Male	Middle school	Spouses and children	Rural	1	<1	1–3	1–2	Not useful	Quite easy	Very Low	Not Very Trustworthy	36
31	68	Female	Vocational school	Spouse	City	2	3–5	1–3	2–3	Quite useful	Average	Quite Low	Average	58
32	65	Male	Middle school	Spouse	City	4	>10	4–6	3–4	Average	Quite easy	Quite High	Average	47

a For patients with multiple chronic diseases, the duration is based on the first diagnosis.

b The Digital Health Literacy Evaluation Scale includes three dimensions: obtaining and assessing digital health information, interaction, and application abilities, with a total of 15 items. The total score ranges from 15 to 75 points, with higher scores reflecting greater digital health literacy. Cronbach’s α = 0.941.

**Table 2:** Frequency of reported chronic diseases (*n* = 32).

Chronic disease	Number of responses	Response percentage (%)	Percentage of cases (%)
Hypertension	24	38.10	75.00
Diabetes	9	14.30	28.10
Hyperlipidemia	2	3.20	6.30
Chronic liver disease	1	1.60	3.10
Cancer	4	6.30	12.50
Chronic respiratory diseases	3	4.80	9.40
Cardiovascular and cerebrovascular diseases	5	7.90	15.60
Neurological diseases	1	1.60	3.10
Gastrointestinal diseases	5	7.90	15.60
Bone and joint diseases	6	9.50	18.80
Others	3	4.80	9.40
Total	63	100.00	196.90

*χ2 = 73.71, df = 10, p<0.0001.*

**Table 3:** Frequency of digital device usage (*n* = 32).

Digital device used	Number of responses	Response percentage (%)	Percentage of cases (%)
Smartphones	32	33	100
Smart speakers	2	2.1	6.30
Digital televisions	17	17.5	53.1
Computers/Tablets	8	8.2	25
Smart wearable devices	4	4.1	12.5
Electronic blood pressure monitors	22	22.7	68.8
Glucometers	10	10.3	31.3
Others	2	2.1	6.3
Total	97	100.00	303.1

*χ2 = 66.71, df = 7, p<0.0001.*

### 2.2 Methods

#### 2.2.1 Development of the interview guide

The Unified Theory of Acceptance and Use of Technology (UTAUT) (32) posits that performance and effort expectations, social influence, and facilitating conditions shape individuals’ willingness to adopt technology, influencing adoption behavior. Additionally, age, gender, experience, and voluntariness of use moderate these relationships. In essence, individuals’ perceptions and attitudes toward digital technology affect their intention to use it, ultimately determining their actual usage. Using the UTAUT model, the research team developed a preliminary interview guide based on a literature review and brainstorming discussions. A pre-interview was conducted with three older adult patients meeting the inclusion criteria (not included in the final study sample). After discussions with clinical nursing experts, the guide was revised and finalized. During formal interviews, researchers adjusted the guide as needed to enhance depth and flexibility.

The finalized interview guide included the following questions: (1) How do you define digital health literacy? (Regardless of the interviewee’s familiarity with the concept, the researcher provided a standardized definition to ensure a shared understanding before further discussion.) (2) What channels do you typically use to obtain health information and manage your health? (3) What are the advantages or concerns of using these channels for health information or management? (4) What difficulties have you encountered in obtaining health information or managing your health through these channels? (5) What factors do you think influence digital health literacy levels? (6) Would you like to improve your digital health literacy? Why or why not? (7) If digital health literacy training were available, what content would you like to learn? Do you have suggestions for the format or content of the training? (8) Do you have any additional thoughts or comments beyond the above topics?

#### 2.2.2 Data collection methods

The study strictly followed the COREQ (Consolidated Criteria for Reporting Qualitative Research) guidelines (33). The COREQ statement for this study is shown in Table 4. Data were collected through face-to-face semi-structured in-depth interviews (34).

**Table 4:** Consolidated criteria for reporting qualitative studies (COREQ): 32-item checklist.

No Item	Guide questions/description	Reply
Domain 1: research team and reflexivity		
Personal characteristics		
1.Interviewer/facilitator	Which author/s conducted the interview or focus group?	Two nursing master’s students and one qualitative research expert
2. Credentials	What were the researcher’s credentials? E.g. PhD, MD	Master
3. Occupation	What was their occupation at the time of the study?	Nursing staff
4. Gender	Was the researcher male or female?	Female
5. Experience and training	What experience or training did the researcher have?	All received professional training
Relationship with participants		
6. Relationship established	Was a relationship established prior to study commencement?	Yes
7. Participant knowledge of the interviewer	What did the participants know about the researcher? e.g. personal goals, reasons for doing the research	Research objectives, methods, and procedures, including recording requirements and confidentiality commitments.
8. Interviewer characteristics	What characteristics were reported about the interviewer/facilitator? e.g. Bias, assumptions, reasons and interests in the research topic	Reasons for conducting the study
Domain 2: study design		
Theoretical framework		
9. Methodological orientation and Theory	What methodological orientation was stated to underpin the study? e.g. grounded theory, discourse analysis, ethnography, phenomenology, content analysis	Data were analyzed using content analysis.
Participant selection		
10. Sampling	How were participants selected? e.g. purposive, convenience, consecutive, snowball	Sampling was conducted through a combination of purposive sampling and snowball sampling.
11. Method of approach	How were participants approached? e.g. face-to-face, telephone, mail, email	Interviews were conducted face-to-face in a designated hospital department meeting room
12. Sample size	How many participants were in the study?	32
13. Non-participation	How many people refused to participate or dropped out? Reasons?	Eight individuals declined participation, citing reasons such as time constraints, lack of interest, and privacy concerns.
Setting		
14. Setting of data collection	Where was the data collected? e.g. home, clinic, workplace	Hospital department meeting room
15. Presence of non-participants	Was anyone else present besides the participants and researchers?	No
16. Description of sample	What are the important characteristics of the sample? e.g. demographic data, date	Demographic information of the participants is provided in Table 1
Data collection		
17. Interview guide	Were questions, prompts, guides provided by the authors? Was it pilot tested?	Yes, after a pilot interview (3 cases)
18. Repeat interviews	Were repeat interviews carried out? If yes, how many?	No
19. Audio/visual recording	Did the research use audio or visual recording to collect the data?	Yes, audio recording
20. Field notes	Were field notes made during and/or after the interview or focus group?	Yes
21. Duration	What was the duration of the interviews or focus group?	20–30 min
22. Data saturation	Was data saturation discussed?	Yes
23. Transcripts returned	Were transcripts returned to participants for comment and/or correction?	Yes
Domain 3: analysis and findings		
Data analysis		
24. Number of data coders	How many data coders coded the data?	59
25. Description of the coding tree	Did authors provide a description of the coding tree?	No
26. Derivation of themes	Were themes identified in advance or derived from the data?	Derived from the data
27. Software	What software, if applicable, was used to manage the data?	Nvivo12.0
28. Participant checking	Did participants provide feedback on the findings?	Yes
Reporting		
29. Quotations presented	Were participant quotations presented to illustrate the themes / findings? Was each quotation identified? e.g. participant number	Yes
30. Data and findings consistent	Was there consistency between the data presented and the findings?	Yes
31. Clarity of major themes	Were major themes clearly presented in the findings?	Yes
32. Clarity of minor themes	Is there a description of diverse cases or discussion of minor themes?	Yes

Before data collection, a research team was formed, consisting of two master’s students in nursing and a qualitative research expert with over 30 years of teaching experience (a professor and graduate supervisor). All team members received systematic training in qualitative research and collaborated on data collection, organization, and analysis.

Before the interviews, researchers obtained approval from the hospital department and participated in daily nursing activities to familiarize themselves with participants’ language habits and cultural backgrounds, fostering trust. They explained the study’s objectives, methods, and procedures, including recording requirements and confidentiality assurances, ensuring participants fully understood the study and provided written informed consent. Interviews were scheduled at the participants’ convenience and lasted 20–30 min. They were typically conducted in a quiet, private consultation room within the hospital to minimize disruptions.

During the interviews, researchers maintained neutrality, guided participants with open-ended questions, used probing techniques flexibly, and encouraged participants to express their genuine views, and participants’ responses were repeated or summarized for clarity. Researchers also observed and recorded participants’ facial expressions and body movements to capture meaningful non-verbal information. After each session, researchers wrote interview diaries to summarize and reflect, continuously improving the interview quality.

#### 2.2.3 Data analysis methods

This study used the triangulation method to analyze data by integrating multiple sources, including research data, researchers, theoretical frameworks, and methodologies. This approach ensured cross-validation of findings, enhancing the study’s reliability and validity (35, 36). Participants were recruited from three hospitals in Anhui, ensuring a representative and diverse sample. Two nursing master’s students transcribed interview recordings within 24 h and invited participants to verify the transcripts for accuracy and authenticity. For data analysis, researchers used Nvivo 12.0 software combined with manual coding. Following the UTAUT, two nursing master’s students independently conducted a content analysis (37), repeatedly coding and comparing data to minimize omissions and bias. A qualitative research expert resolved any discrepancies through discussion, ensuring consensus before finalizing the results.

The data analysis followed these steps: (1) Researchers repeatedly listened to recordings and thoroughly reviewed transcripts, maintaining neutrality for an in-depth understanding of the data.(2) Identifying and extracting meaningful statements related to factors influencing digital health literacy as the smallest unit of analysis.(3) Performing open coding to extract concepts from meaningful statements.(4) Grouping related codes into themes and sub-themes, supported by examples to generate the research findings. The main interview results are summarized in Table 5.

**Table 5:** Summary of semi-structured interview findings on digital health literacy in older adults with chronic diseases.

Category	Questions	Findings (Number)
Background: importance and necessity	(1) How do you define digital health literacy?	8 participants had heard of it but misunderstood its meaning.24 had never heard of it. After a standardized explanation, all 32 acknowledged its importance. Facilitates health management (16). Recognize as a necessity in the digital era and worth learning (19).
Experience (Analyzed by Age/Gender)	(2) What channels do you typically use to obtain health information and manage your health?	Devices Used: Smartphones (32), Digital TV (17), Smartwatches (4), Smart speakers (2), Computers/Tablets (8), Electronic blood pressure monitors (22), Blood glucose monitors (10), Pulse oximeters (1), Home-use ventilators (1). Information Sources: Baidu (24), WeChat (22), Built-in mobile search engines (9), Toutiao/Sina (7), Short-video platforms (12), Haodaifu Online (3), Xiaohongshu (3), WPS for blood glucose recording (1). Content/Features Accessed: Disease knowledge (26), Medication guidance (19), Dietary advice (21), Exercise recommendations (15), Hospital appointment booking (26), Online consultations (6).
Performance expectation: benefits and barriers	(3) What are the advantages or concerns of using these channels for health information or management?	Benefits: Expanded disease knowledge (26), Diverse health information sources (13), Multiple access channels (21), Convenience in medical consultations (11), Time and cost savings (12), Transparency in medical and financial information (9), Increased health awareness (14), Improved lifestyle habits (7), Easier condition monitoring (5). Concerns: Personal data leaks (12), Financial security risks (8), Lack of digital skills (18), Susceptibility to misleading commercial promotions (11), Frequent spam calls (7).
EFFORT expectation: ease of use	(4) What difficulties have you encountered in obtaining health information or managing your health through these channels?	Vision/hearing impairment (9), Low education level/limited comprehension (14), Complex technical operations (22), Inconsistent information quality (16), Privacy and security concerns (7).
Social Influence and facilitating conditions	(5) What factors do you think influence digital health literacy levels?	Facilitating Factors: Widespread smartphone adoption (18), Societal progress (10), Guidance from healthcare professionals and volunteers (18), Information shared by relatives and friends (8). Barriers: Digital technology obstacles (14), Cognitive decline and poor adaptability (7), Frequent updates and complex functions (9).
Voluntary use	(6) Would you like to improve your digital health literacy? Why or why not?	Willing to improve: Recognized as essential for digital society, perceived benefits, and convenience, willing to learn (16). Unwilling to improve: Age-related reluctance (6), Reliance on family members (3), Vision impairment (1), Lack of energy (3), Difficulty retaining learned skills (8), Time constraints (4). No need for improvement: Current skills sufficient (1).
Other considerations	(7) If digital health literacy training were available, what content would you like to learn? Do you have suggestions for the format or content of the training?	Preferred Topics: Accessing authoritative sources (14), Technical operation skills (13), High practicality (4). Suggestions: Simplified operations (10), Community-based training (9), In-person lectures (8), Taught by healthcare staff (8), Regular sessions (4), Flexible scheduling (2), Personalized guidance (9), Combination of online/offline learning (6), Long-term, fixed-location training (4).
Additional feedback	(8) Do you have any additional thoughts or comments beyond the above topics?	Responses: No additional input (11). Emphasized personal choice and respect for individual willingness (5). Called for stronger regulatory oversight (3). Preferred pure health education without commercial promotion (2). Hope for real implementation of digital health literacy initiatives (3). Other unrelated comments (8).

## 3 Results

Based on the UTAUT model, factors influencing digital health literacy in older adult patients with chronic diseases were classified into two main themes and nine sub-themes, as outlined below.

### 3.1 Theme 1: driving factors

#### 3.1.1 Accessibility of digital health resources

##### 3.1.1.1 Diversified channels for health information acquisition

Older adult patients with chronic diseases access health information through various digital platforms, including search engines, social media, news websites, and health-related mobile applications. Most respondents reported frequently using smartphones to obtain health knowledge, seek medical consultations, and manage chronic conditions. For instance, N2 and N3 stated, “WeChat, Baidu, Toutiao, Douyin, Haokan Video, and Xiaohongshu are all very convenient.” N7 and N9 added, “Now, as soon as I open my phone, I can access Sina News and Alipay—it’s very convenient.” N11 and N14 noted, “In the past, when I had a problem, I did not know who to ask. Now, I can just search on Baidu, saving me trips to the hospital.”

Some respondents also relied on health-related mobile applications for professional information and short video platforms for wellness education. For example, N13 stated, “I enjoy listening to ‘Ximalaya’ and watching Douyin.” N16 mentioned, “The nurses in my department recommended the hospital’s official WeChat account.” N21 added, “I use WPS to record my blood sugar levels.”

These findings suggest that older adults are increasingly turning to digital sources for health information. Their platform preferences are influenced by ease of use, content presentation, and specific health information needs. Short video platforms, for instance, offer accessible and easily digestible health education, reducing cognitive burden while enhancing engagement. Additionally, some respondents preferred official sources, such as hospital WeChat accounts or professional health applications, to ensure credibility.

##### 3.1.1.2 Expanding access to digital health resources

For some older adults, limited access to offline medical services poses a challenge, whereas digital health resources help bridge this gap. Many respondents reported using online sources to obtain information on diet, exercise, and medication, as well as digital health tools (e.g., electronic blood pressure monitors and smart wristbands) to track their health. These technologies overcome traditional constraints of time and location, allowing patients to access guidance anytime, anywhere, and promoting proactive health management.

For example, N24 stated, “My home is far from the hospital, and getting there is difficult.” N30 mentioned, “Doctors from the town visit our village for check-ups, but they leave right after, making follow-ups difficult.” N7 and N31 shared, “We can check medication side effects at any time and learn how to recover after chemotherapy.” N26 demonstrated, “(Showing the device) I use a mobile app and a smart wristband to monitor my heart rate, breathing, and step count at any time.” N14 and N18 noted, “We have electronic blood pressure monitors and glucose meters at home, so we can measure them anytime—they are very practical.”

These findings underscore the value of digital health resources as a vital supplement in chronic disease management. For patients in remote areas or those with mobility limitations, online health information and digital monitoring devices enhance their ability to make informed healthcare decisions. However, individual differences in digital health literacy remain evident. While information acquisition is now more convenient, challenges persist in filtering and understanding health information, as well as in mastering digital health tools.

#### 3.1.2 Value perception and management needs

##### 3.1.2.1 Perceived benefits

Most respondents believed that improving digital health literacy benefits health information access, disease awareness, and chronic disease management. Many emphasized that higher digital health literacy allows them to take a proactive approach to understanding their health, reducing uncertainty in decision-making. Some noted that lacking digital health literacy makes it difficult to independently obtain reliable information, often leading to confusion when facing health issues. For instance, N1 stated, “Improving digital health literacy is essential for better understanding one’s health.” N2 commented, “Having no digital health literacy is unacceptable… Otherwise, when problems arise, I’ll be completely lost.” N5 added, “It is crucial for chronic disease management and must be taken seriously.” These findings suggest that older adults who perceive greater benefits from digital health literacy are more likely to adopt health-related behaviors.

##### 3.1.2.2 Meeting health management needs

Illness experience drives patients to actively seek health information and prioritize health management. Several respondents reported relying on digital health resources after being diagnosed with a chronic disease to obtain information on diet, exercise, and medication management. N5 and N31 (colon cancer patients) stated, “After falling ill, I pay special attention to health information.” Some noted that when doctors provided limited guidance on dietary restrictions or medications, they supplemented their knowledge online.

Additionally, some respondents highlighted using digital tools to optimize caregiving for family members, emphasizing that digital health literacy influences both personal and family healthcare. N15 noted, “My father, over 90 years old, is frequently hospitalized… I need to learn how to care for him.” N16 stated, “My mother has been bedridden for years; I learn caregiving techniques online.” These findings indicate that older adults’ need for digital health literacy is closely linked to their health management demands.

#### 3.1.3 Family economic and social benefits

##### 3.1.3.1 Optimizing family economic efficiency

Digital healthcare simplifies medical procedures, reducing the need for family support during visits and streamlining insurance reimbursements, thereby easing financial and time burdens. N13: “My child books appointments online, so I can go alone, letting them focus on work.”N20: “Insurance reimbursements can be processed via phone, avoiding repeated trips.” N31: “Phone-based reimbursement processes reduce my children’s worries and give them peace of mind.”

Digital health resources improve family economic efficiency by streamlining medical procedures, reducing healthcare costs, and facilitating insurance reimbursement. Many respondents reported that online appointment booking and digital insurance claims allowed them to complete medical processes independently, reducing the burden on family members. For instance, some noted that scheduling appointments via mobile phones minimized disruptions to their children’s work, while digital insurance operations reduced hospital visits and expedited reimbursements. This alleviates financial strain and enhances patient independence. N13 and N31 shared, “We book appointments on our phones and go alone, so our children can focus on work.” N20 stated, “Medical insurance claims can be processed via mobile phone, eliminating repeated hospital visits.”

##### 3.1.3.2 Enhancing healthcare experience and medical transparency

Digital healthcare improves older adults’ medical experiences by reducing wait times, increasing accessibility, and enhancing transparency. Several respondents noted that registering for appointments previously required long queues, whereas online registration now minimizes wait times and enhances convenience. Additionally, online consultations provide valuable access to medical advice, particularly for those in remote areas, preventing unnecessary travel and treatment delays. N13 and N31 stated, “Online appointment booking eliminates long queues, making it very convenient.” N15 added, “I can consult specialists from other cities via mobile phone, which saves both effort and money.”

Moreover, digital healthcare has increased transparency in medical expenses. Some respondents noted that digital applications display medication records, medical tests, and cost details, allowing them to better track their spending and reducing anxiety caused by information gaps. N29 explained, “I can check my phone for medication and test records, so I know exactly where my money goes.” Others reported using online resources to clarify medication instructions, improving adherence. N26 and N32 stated, “I could not understand the doctor’s instructions, so I looked up how to take the medication online.” These findings suggest that digital healthcare enhances both the medical process and patient autonomy in decision-making.

#### 3.1.4 Social network support system

##### 3.1.4.1 Support from family members

Family plays a crucial role in older adults’ adoption of digital health tools, providing device access, technical guidance, and emotional support. Many respondents stated that their children or relatives helped them acquire and use digital health technologies. For instance, N23 said, “My children bought me a blood pressure monitor and a glucose meter and remind me to measure regularly.” This suggests that intergenerational support significantly influences digital health adoption.

##### 3.1.4.2 Encouragement from peer groups

Peer groups, particularly those with shared medical conditions, provide motivation and credibility in adopting digital health technologies. Many older adults noted that after observing peers successfully using digital health tools, they became more inclined to learn. Some were inspired to use mobile-based appointment booking after seeing hospital roommates navigate the system. N28 remarked, “The patient next to me is over 80 and can book appointments on a phone. I’m in my 60s—this motivated me to learn; I cannot fall behind.” These findings suggest that peer role modeling drives behavioral change.

##### 3.1.4.3 Assistance from healthcare institutions

Community hospitals, health centers, and pharmacies play a vital role in bridging digital health gaps among older adults. Some respondents reported seeking help from community healthcare providers when struggling with digital tools, such as glucose meters. N3 and N8 stated, “The community hospital and pharmacy are close, so when I have trouble using my glucose meter, I ask for help.” Additionally, some noted that outpatient volunteers and hospital staff provided guidance on online appointment booking, making digital healthcare services more accessible. N30 said, “An outpatient volunteer taught me how to book appointments online—it was very helpful.” These findings highlight the importance of institutional support in facilitating digital health adoption.

### 3.2 Theme 2: constraints

#### 3.2.1 Cognitive blind spots and misunderstandings

##### 3.2.1.1 Cognitive blind spots

Most respondents were unaware of digital health literacy, demonstrating low recognition and delayed comprehension. Many stated they had never heard of it or hesitated when asked related questions. For example: N8: “I do not understand. I cannot comprehend it.”N10, N14: “Um… (pause) Not very clear.” N21: “I do not know about it. This is the first time I’ve heard of it.” N26: “(Pause, thinking) … No (never heard of it).”

This lack of awareness may stem from the rapid advancement of digital technology, making it difficult for older adults to keep up. Traditional health education has mainly focused on disease management, with little emphasis on digital skills. Additionally, some older adults resist new technologies or have low self-efficacy.

##### 3.2.1.2 Misunderstandings

Some respondents (8 individuals) had heard of “digital health literacy” but misunderstood it, often associating it with smartphone proficiency, online shopping, or artificial intelligence. Many equated it with mobile phone usage and accessing health information, overlooking its core aspects, such as evaluating digital health information and making informed decisions. These misconceptions may arise from limited information sources and the lack of targeted health education. Additionally, personal experiences and social environments shape learning and cognition. Older adults mainly exposed to digital services in daily life are more likely to equate digital health literacy with general tech skills. For example: N29: “It’s just about using your phone well and caring about health knowledge.” N23: “Isn’t it artificial intelligence and robots making life easier for older adults?” N18 (a rural-to-urban migrant living in Guangzhou for over 10 years): “The gap in digital development between small towns and big cities is too large.”

#### 3.2.2 Basic skills and digital adaptation challenges

##### 3.2.2.1 Weak basic skills

Many older adults struggle to interpret health information due to low education levels, limited literacy, and a lack of medical knowledge. Some relied on voice or video content because they were illiterate or unfamiliar with input methods. Those without formal education found complex medical terminology difficult to understand or remember, even when doctors provided detailed explanations. For example: N8: “I do not know how to type. I just watch videos and send voice messages.” N20: “I’m from the countryside. I had many siblings… I dropped out of school early and have little education.” N26: “I cannot understand or remember the professional content explained by doctors.”

Limited exposure to digital media and technology further contributed to unfamiliarity, leading to anxiety and avoidance. Some respondents mentioned that frequent updates in smart devices made adaptation challenging, triggering resistance. Physiological decline, such as deteriorating vision, hearing loss, and cognitive decline, further exacerbated difficulties, reducing willingness to use digital tools. N2: “There are too many functions. Before I learn one, it updates again. It’s frustrating.” N22: “I’m too old to learn and do not want to anymore.” N6, N10: “My eyesight is poor, and I worry about relying too much on smartphones.” N13: “I cannot use smartphones as well as children. I feel outdated.”N17, N25: “My memory is poor. I forget what I learned—I’m old.”

Due to weak digital skills, older adults face multiple obstacles when accessing health information, particularly when using self-service machines, booking appointments online, or searching for health content. Many struggle with complex digital healthcare processes and feel overwhelmed. Rural residents, in particular, have limited exposure to digital technology, leading to a lack of confidence. Fearing errors or damaging devices, they often avoid usage altogether, exacerbating health information inequality. N13, N19: “I cannot use self-service machines or figure out how to book an appointment on my phone.” N14: “WeChat appointment booking and online consultations are too complicated. I cannot find the right section.”N22: “I do not know how to search for health information. If I cannot find it, I just give up.”N24, N30: “We do not have these devices in rural areas. I do not dare use them—I’m afraid of breaking them.”

##### 3.2.2.2 Digital adaptation barriers

Older adults often struggle with digitalized healthcare procedures, negatively impacting their medical experience. Some reported difficulties with online appointment booking and digital payments, sometimes resulting in financial losses. However, after guidance from medical staff or family members, some gradually adapted and were able to book appointments independently. N14: “I messed up online booking and lost money.”N24: “At first, I did not know how to use it, but after a nurse taught me, I could book my own follow-ups.”N30: “I had never seen it before and could not figure it out. A nurse had to help me.”

Many older adults distrust online consultations, as they lack the sense of reality and personal connection of face-to-face interactions. Most respondents felt that online consultations could not replace in-person visits, particularly when choosing a doctor. They preferred recommendations from acquaintances or existing doctor-patient relationships over online information. Additionally, some doubted the credibility of online doctors, believing digital platforms could not accurately assess medical professionals’ expertise, reinforcing their preference for in-person visits. N8, N22: “You cannot see the doctor online, so it does not feel reassuring.”N11: “How do I know who’s really on the other side? My home is near the hospital, and I trust Dr. X because I know him personally. If I need a referral, I’d rather have him recommend someone.”N21: “I check doctors’ credentials online, but I still prefer visiting the hospital—it feels more reliable.”

Adaptability to digital health tools varies depending on their complexity. Older adults could easily use simple devices like electronic blood pressure monitors and smart wristbands but found more complex tools, such as blood glucose monitors, difficult to operate. This reduced their willingness to use them. Additionally, some questioned the accuracy and reliability of digital health devices, further limiting their use. N1: “With an electronic blood pressure monitor, you just strap it on and press a button. It tells you if your pressure is too high or low, and you adjust your medication accordingly.”N2: “I can use wrist and arm blood pressure monitors, but I cannot handle the blood glucose monitor.”N4, N11: “Once the wristband is set up, it works fine—no issues.”N5: “I often check my blood pressure digitally, but I find blood glucose monitors too troublesome. I feel they are inaccurate and unreliable.”N17: “There’s nothing difficult about using an electronic blood pressure monitor.”

#### 3.2.3 Social and psychological constraints

##### 3.2.3.1 Multiple role conflicts

Older adult patients must balance family responsibilities, social activities, and health management, which reduces their willingness and capacity to engage in digital health management. Many interviewees reported that caring for grandchildren, older adult parents, or managing household duties made it difficult to invest time in learning digital health skills. Some prioritized their children’s or grandchildren’s education at the expense of their health. N8: “My grandson is taking the college entrance exam next year. Everything revolves around him, and I have no time to focus on my minor ailments.”N15: “I live with my 90-year-old father and have to take care of him.” N20: “I have cared for my younger siblings, my children, and now my grandchildren.” N22: “I care for my 80-year-old mother, do the grocery shopping, cook, and manage house renovations. My child is at work and cannot help.” Additionally, some respondents indicated that active participation in social activities, such as attending senior university courses or community events, reduced their opportunities to access digital health resources. N3: “I take classes at a senior university and often attend gatherings.” This phenomenon suggests that many older adult patients voluntarily assume intergenerational responsibilities, prioritizing family obligations over personal health management. A strong sense of familial duty may limit their willingness to engage with digital health tools.

##### 3.2.3.2 Social image concerns

When obtaining and sharing digital health information, older adult patients exhibit heightened sensitivity to their social image, making them cautious about sharing health-related content. Some fear that sharing unverified information could mislead others, cause misunderstandings, or damage their reputation. N3: “I tried kudzu root powder (a health supplement) and found it effective, so I shared it on social media. However, I hesitated to actively recommend it, fearing others might think I was selling medicine.” This self-restraint reflects their desire to maintain social harmony and avoid being perceived as having commercial motives.

Moreover, family members’ interventions influence their behavior. Some respondents noted that after purchasing health products online, they wanted to recommend them to friends but were discouraged by their children, who feared potential risks. N10: “I bought medicine for back pain on ‘Kuaishou’ (a short video platform) and found it helpful. I shared it in my social media groups, but my child advised against recommending it, fearing allergic reactions or unintended consequences.” Such external constraints reinforce older adult patients’ reluctance to share health information, reducing their participation in digital health discussions.

##### 3.2.3.3 Health information avoidance

Some older adult patients deliberately avoid health information to reduce anxiety and emotional distress. This avoidance manifests in both emotional and social dimensions. At the emotional level, some interviewees felt that excessive focus on health issues increased their anxiety, prompting them to avoid health-related content. N22: “I do not want to look at these things. The more I see, the more annoyed I feel.” At the social level, health discussions are sometimes seen as taboo among older adult social circles. Some respondents reported receiving negative reactions when sharing health information. N26: “I shared health information with a friend, and they said I was cursing them. They joked that I was ‘afraid of dying,’ so I stopped bringing it up.” This suggests that in some social contexts, discussing health matters may be culturally sensitive, leading older adult patients to avoid digital health interactions.

#### 3.2.4 Issues with health information quality

##### 3.2.4.1 Severe information overload

Many interviewees found the vast quantity and inconsistent quality of online health information overwhelming. Some noted that complex webpage layouts and excessive advertisements made it difficult to focus. N9: “There’s too much text, the font is too small, and there are too many ads. I do not want to look at it.” N29: “Online health information is chaotic and disorganized. It’s hard to distinguish truth from falsehood.” Additionally, some content prioritizes marketing over education, promoting medications rather than providing reliable health knowledge. N20, N24: “The information is superficial. In the end, it’s all about selling medicine rather than educating people.” Information overload increases cognitive burden, making it harder for older adult patients to filter and evaluate content, ultimately limiting their access to valuable health information.

##### 3.2.4.2 Low credibility of information

Many respondents were skeptical about the authenticity of digital health information, particularly when different sources provided contradictory explanations for the same issue. N11: “For the same question, some sources say one thing, others say the opposite. I do not know which to trust.” Inconsistent information led to distrust, with some interviewees criticizing self-proclaimed experts who spread exaggerated or misleading health claims. N6: “There’s so much information online, some even claim to cure cancer—what nonsense.” N13: “Many fake experts read a few books and start spreading misinformation. In the end, it’s all about selling medicine.”

##### 3.2.4.3 High degree of information homogeneity

Some respondents observed that digital health content was repetitive, leading to a perception that it lacked novelty or in-depth guidance, which diminished their interest over time. N5: “It’s all the same repetitive content. I cannot find what I need.” N21: “I used to read it often, but eventually realized that everything is superficial and repetitive, so I lost interest.” Content homogeneity discourages continued engagement and hinders digital health literacy. Enhancing personalization, relevance, and depth of health information may improve user engagement.

#### 3.2.5 Perceived data security risks

Although older adult patients are generally aware of privacy and financial security risks, their limited digital skills and knowledge of online security constrain their ability to protect themselves. Instead, they rely on personal experience and trusted acquaintances to manage risks. Some interviewees avoided unfamiliar links and only trusted information shared by relatives and friends. N15: “I only read content shared by people I know. I do not trust unfamiliar links.” Some adopted a “minimalist” approach, such as not linking bank cards or using only small amounts for mobile payments to minimize potential losses. N22: “I’m not worried. I do not link my bank card—I just use the few hundred yuan my child transfers to me via WeChat.”

However, heightened risk perception affected their online interactions. Some expressed willingness to share illness experiences but refrained from online discussions due to privacy concerns. N5: “I will not post online. I’m worried about malicious websites collecting my data.” N13, N28: “I generally do not participate online, leave comments, or forward posts.”N15: “I do not engage with links or voting activities from strangers.”N24: “I accidentally clicked on something before, and my phone number was leaked. Now I get marketing calls every day.”N29, N31: “I do not dare to grant permissions. My WeChat is linked to my salary account, and my pension is there. I’m afraid of being scammed.” Overall, high sensitivity to data security risks discourages older adult patients from engaging with digital health resources, limiting their ability to obtain and share health knowledge, and ultimately hindering digital health literacy development.

## 4 Discussion

### 4.1 Individual level: interaction between subjective concepts and objective conditions

#### 4.1.1 Cognitive blind spots and misunderstandings of digital health literacy

This study found that older adult patients have cognitive blind spots and misunderstandings regarding digital health literacy. They often equate it solely with browsing health information on smart devices while neglecting key competencies such as information filtering, decision-making, and digital chronic disease management. This may stem from the fact that digital health literacy, as an emerging concept, has yet to gain widespread recognition in China. Additionally, older adult patients primarily use digital devices for socialization and entertainment, paying little attention to health-related functions. Consequently, their ability to utilize digital health services remains significantly limited (38, 39).

From the microsystem perspective of the social-ecological system theory, aging and chronic disease progression lead to physiological and cognitive decline, further restricting older adult patients’ access to the internet and digital health skills. When attempting to learn these skills, low self-efficacy and prior failures often result in resistance and negative attitudes, with some believing they “cannot learn” or “do not want to learn.” Others hold pessimistic views such as “I will not live much longer” or “Digital technology is for young people.” Consistent with previous studies (24, 40), negative aging attitudes not only heighten social isolation and loneliness but also exacerbate anxiety about digital technology, hindering its adoption. Conversely, research indicates that older adult patients with higher self-efficacy and positive attitudes toward aging are more inclined to explore and learn digital health skills, thereby enhancing their digital health literacy (41, 42). To address this, health education should guide older adult patients in developing a correct understanding of aging, boosting their confidence and adaptability in digital environments to improve digital health literacy.

Moreover, this study found that some older adult patients have limited medical knowledge. Due to differences in doctor-patient perspectives and constrained consultation time, they often struggle to fully comprehend medical advice. As a result, they turn to digital platforms for supplementary information. This aligns with previous research, which suggests that the complex needs of chronic disease management drive patients to actively seek digital health resources, improving their digital health literacy (43). Medical institutions should offer personalized health education and continuously assess older adult patients’ physical and mental conditions to enhance their acceptance and application of digital health services.

#### 4.1.2 Lack of digital security awareness and insufficient preventive measures

This study found that older adult patients generally lack awareness of digital security risks when using digital health services, consistent with previous research (44). Some have experienced online fraud or misinformation, leading to distrust or even rejection of digital health platforms in favor of traditional offline methods. Others oversimplify cybersecurity, believing that avoiding unfamiliar links or not linking their bank accounts is sufficient protection while overlooking more complex threats, particularly those arising from social interactions with acquaintances.

From the microsystem perspective of the Socio-Technical Environment (SET) theory, older adult patients’ risk perception and decision-making abilities are shaped by cognitive capacity, education, and past experiences. Many lack digital security education and struggle to assess health information credibility. Previous studies suggest that digital risk assessment skills directly influence the acceptance and frequency of digital health service use among older adult patients (10). To mitigate these risks, targeted digital security education is essential. Strengthening critical thinking and risk-avoidance skills can enhance trust in digital health services, ensuring safer and more effective engagement with digital platforms.

#### 4.1.3 Dual constraints on information acquisition and active application

This study found that some older adult patients passively receive health information, often encountering it incidentally on smartphones rather than actively seeking it. Some prioritize authoritative sources and validate information through personal experience, peer verification, and cross-referencing multiple channels. However, cognitive limitations, technology acceptance, and information accessibility constrain their ability to acquire digital health information (44, 45). Research suggests that diverse information sources significantly enhance health literacy (46, 47). Nevertheless, some older adult patients overly rely on hospital-affiliated public accounts while dismissing alternative sources, limiting their information intake.

Additionally, older adult patients with multiple chronic diseases are more prone to health information avoidance, deliberately evading disease-related content to reduce psychological stress. This aligns with previous findings (48, 49), which indicate that concerns about disease progression or a lack of coping strategies drive such avoidance. However, avoiding health information diminishes motivation to acquire health knowledge, ultimately compromising disease management.

Furthermore, China’s digital health sector lacks unified regulatory standards. Some search engines and short-video platforms engage in “paid ranking” practices, resulting in inconsistent information quality and making it difficult for users to identify reliable sources. This study found that older adult patients with limited medical knowledge often disregard complex health information rather than translate it into practical chronic disease management strategies. To address these challenges, regulatory authorities should strengthen oversight of digital health content, optimize information presentation, and provide evidence-based training. Enhancing older adult patients’ ability to discern credible information will enable them to translate health knowledge into effective chronic disease management behaviors.

### 4.2 Interpersonal level: the dual role of family support and social interaction

In the social-ecological systems theory, the interpersonal level emphasizes the interaction between individuals and their family and social support networks in shaping digital health literacy. This includes family and social support as well as the reciprocal effect of digital technology.

#### 4.2.1 Dual challenges of multiple role conflicts

In modern society, older adult patients often assume multiple roles, including chronic disease managers, family caregivers, and social participants. In traditional Chinese family values, older adult patients regard maintaining their health as part of their familial responsibilities. To avoid becoming a burden on their children, they actively learn digital technologies to better manage chronic diseases, thereby enhancing their self-efficacy and sense of value. Additionally, some older adult patients, when acting as caregivers, actively seek and apply health information to meet caregiving needs.

However, the rapid updates of digital devices and their operational complexity require significant time and effort to learn, while traditional family values compel older adult patients to prioritize family needs over their chronic disease management. This role conflict and resource constraint limit their ability to improve digital health literacy. This study found that in multigenerational households, older adult patients often bear responsibilities such as household chores and caring for grandchildren, restricting their time for acquiring digital health skills. In contrast, those in relatively independent family structures tend to have greater autonomy in digital health management.

Therefore, healthcare professionals should encourage older adult patients to enhance their proactive health awareness, promote family communication, and leverage community support to help them balance family responsibilities with personal health management. Additionally, regular and multi-level digital skills training should be conducted to improve their digital health competencies, reducing the social stigma associated with learning and ultimately enhancing their digital health literacy.

#### 4.2.2 Influence of social support networks

The trust relationships that older adult patients establish within peer groups or among fellow patients facilitate the acceptance and sharing of health information. Health information shared within familiar social circles is perceived as filtered and validated, aiding comprehension and communication. Such interactions provide demonstrative and motivational effects. However, the prevalence of online fraud has heightened older adult patients’ concerns about data privacy and financial security, reinforcing their reliance on trusted social circles.

Older adult patients’ interactions on social platforms (e.g., WeChat) not only serve as important channels for obtaining health information but also promote information sharing. Social support networks help bridge the information gap for older adult patients, aligning with previous research findings that emotional, informational, and instrumental support within social networks alleviate stress and improve physical and mental well-being (50). These networks are thus key factors influencing digital health literacy (41).

Additionally, older adult patients’ sensitivity to social image makes them cautious about publicly sharing health information, particularly regarding disease privacy and health-related decisions. While some older adult patients wish to share health information or disease experiences to help others, they fear providing inaccurate information that might mislead others or be misinterpreted as promotional activity. Consequently, they adopt an avoidant attitude toward health information sharing. Furthermore, within the context of traditional Chinese culture, health issues are often considered sensitive or private topics. Discussing health information may be perceived as inauspicious or as touching upon life-and-death matters, potentially disrupting social harmony and hindering health information exchange and dissemination.

#### 4.2.3 The need for improved “digital feedback” functions

This study found that emotional support and practical assistance from family members—such as guidance in accessing and interpreting health information—enhance older adult patients’ confidence and ability to use digital health technologies. Older adult patients lacking family emotional and technological support are prone to experiencing “digital loneliness” and technology-related anxiety. Previous research suggests that younger generations teaching older adult patients digital skills, known as “digital reciprocity,” is a crucial measure for bridging the digital divide (51). This process fulfills older adult patients’ desire for digital learning and helps alleviate their digital technology anxiety (52).

However, disparities arise due to variations in family structures, children’s educational backgrounds, and digital competencies. Similar to previous findings (51), harmonious intergenerational relationships, higher family education levels, and better economic conditions improve the effectiveness of digital reciprocity. Positive intergenerational interactions also enhance emotional communication within families, strengthening older adult patients’ sense of belonging and security, fostering their enthusiasm for learning, and improving their ability to assimilate knowledge. This facilitates their adaptation to digital transformation and access to accurate and efficient health information. Nevertheless, this study also found that some older adult patients overly rely on family members, leading to information biases or excessive dependence, thereby reducing their ability to independently obtain and evaluate digital health information. This aligns with prior research indicating that most older adult patients are passive recipients of digital health information (10).

While family support can enhance older adult patients’ acceptance of digital health tools, the long-term effectiveness of “digital reciprocity” is limited due to the time and energy constraints of younger generations. Consistent with previous findings (51), the support provided by younger generations tends to be intermittent, fragmented, and sporadic, making it difficult to meet older adult patients’ needs for continuous and systematic learning. Therefore, social forces should be mobilized to encourage community and healthcare institutions to offer sustained and structured training programs to supplement the gaps in family education.

### 4.3 Social level: accessibility of digital health resources

This study found that older adult patients in rural or remote areas have relatively low levels of digital health literacy, which aligns with previous findings that low-income older adult groups often face significant digital health disparities due to economic and technological limitations (53). The disparity in social and economic development between urban and rural areas, uneven digital infrastructure, and imbalanced healthcare resource allocation restrict older adult patients’ ability to access high-quality digital health services (54).

In recent years, driven by policy support, technological advancements, and increasing social demand, China’s digital healthcare market has expanded rapidly. To enhance the accessibility of digital healthcare resources and improve patient experiences, China has been actively promoting the development of digital hospitals, upgrading primary healthcare digitalization, advancing internet hospitals, and implementing digital health insurance management initiatives (55).

Compared to traditional health information acquisition methods (such as advice from relatives and friends, television, and newspapers), digital health resources, with their open and shared characteristics, transcend time and space limitations, making information dissemination more diversified, personalized, and interactive. Older adult patients can independently search for health resources based on their needs, reducing acquisition costs and enhancing their health management capabilities (56). This study found that older adult patients’ perceived usefulness and ease of use of digital health information positively influence their willingness to accept and utilize digital health resources, consistent with prior research (57). The adoption of digital health resources can improve health lifestyles and self-management of chronic diseases.

However, although older adult patients tend to trust digital health information provided by peers, their limited communication range may lead to inconsistent information quality, potentially affecting health decisions. At the societal level, national policies and healthcare institutions are actively promoting digital healthcare transformation while improving the accessibility and age-friendliness of digital health services. Therefore, it is recommended that healthcare professionals integrate both online and offline approaches to provide systematic guidance for older adult patients, enhancing their ability to filter and evaluate health information.

## 5 Limitations

This study was conducted in three tertiary hospitals in Anhui Province, where access to digital medical resources is greater than in community or rural healthcare settings. This may limit the generalizability of the findings to older adults in under-resourced areas. Future research should adopt a multi-center approach, including primary care and rural hospitals, to better capture variations in digital health literacy across different healthcare settings.

Additionally, participants were required to own or have access to digital devices and use them independently or with assistance. While this ensured relevance to the study’s focus, it may have excluded individuals with very low digital literacy or those unwilling to engage with digital tools, introducing potential selection bias. Future research should examine the perspectives of digitally excluded older adults to provide a more comprehensive understanding of digital health disparities.

This study used a mixed-methods approach, combining qualitative interviews with quantitative demographic analysis and digital health literacy scale scores. Although this offered some degree of triangulation, the reliance on self-reported interview data may have introduced social desirability bias. Moreover, researchers’ prior knowledge and assumptions could have influenced data interpretation. To strengthen research credibility, future studies should expand quantitative components, such as longitudinal tracking of digital literacy trends or large-scale surveys. Incorporating additional qualitative perspectives from family members and healthcare providers, along with expert review and multiple coding strategies, could further enhance analytical rigor.

## 6 Conclusion

Digital health literacy is essential for managing health in the digital age. This study found that older adult patients with chronic diseases generally have low digital health literacy, characterized by cognitive gaps, comprehension difficulties, and limited ability to acquire, evaluate, and apply digital health information. These challenges stem from multiple factors, including physical and mental health conditions and social support systems.

To address this issue, healthcare professionals should enhance education and awareness programs, regularly assess patients’ physical and mental well-being, and encourage the use of digital tools for chronic disease management. Broader societal efforts are needed to develop a supportive “digital feedback” system, improve access to digital health resources, and foster an inclusive digital health environment. Through collective action, older adult patients can better utilize digital health technologies, ultimately improving health outcomes and quality of life.

## Data Availability

The datasets presented in this article are not readily available because the datasets presented in this article are not readily available because the dataset generated and analyzed during the current study contains personal health information from older adult patients with chronic diseases, and its use is subject to strict confidentiality agreements. Due to privacy and ethical concerns, the data cannot be made publicly available. Access to the dataset is restricted and available only upon reasonable request, with approval from the ethics committee of the participating institutions, in compliance with applicable data protection regulations. Requests to access the datasets should be directed to XY, 0700013@bbmu.edu.cn.
